# Functional genomics of a *Spiroplasma* associated with the carmine cochineals *Dactylopius coccus* and *Dactylopius opuntiae*

**DOI:** 10.1186/s12864-021-07540-2

**Published:** 2021-04-06

**Authors:** Arturo Vera-Ponce León, Marian Dominguez-Mirazo, Rafael Bustamante-Brito, Víctor Higareda-Alvear, Mónica Rosenblueth, Esperanza Martínez-Romero

**Affiliations:** 1 Programa de Ecología Genómica, Centro de Ciencias Genómicas, Universidad Nacional Autónoma de México, Cuernavaca, Mexico; 2 Present Address: Faculty of Biotechnology, Chemistry and Food Science, Norwegian University of Life Sciences, 1433 Ås, Norway; 3 Present Address: School of Biology, Georgia Institute of Technology, Atlanta, GA USA

## Abstract

**Background:**

*Spiroplasma* is a widely distributed endosymbiont of insects, arthropods, and plants. In insects, *Spiroplasma* colonizes the gut, hemolymph, and reproductive organs of the host. Previous metagenomic surveys of the domesticated carmine cochineal *Dactylopius coccus* and the wild cochineal *D. opuntiae* reported sequences of *Spiroplasma* associated with these insects. However, there is no analysis of the genomic capabilities and the interaction of this *Spiroplasma* with *Dactylopius.*

**Results:**

Here we present three *Spiroplasma* genomes independently recovered from metagenomes of adult males and females of *D. coccus*, from two different populations, as well as from adult females of *D. opuntiae*. Single-copy gene analysis showed that these genomes were > 92% complete. Phylogenomic analyses classified these genomes as new members of *Spiroplasma ixodetis*.

Comparative genome analysis indicated that they exhibit fewer genes involved in amino acid and carbon catabolism compared to other spiroplasmas. Moreover, virulence factor-encoding genes (i.e., *glpO*, *spaid* and *rip2*) were found incomplete in these *S. ixodetis* genomes. We also detected an enrichment of genes encoding the type IV secretion system (T4SS) in *S. ixodetis* genomes of *Dactylopius*. A metratranscriptomic analysis of *D. coccus* showed that some of these T4SS genes (i.e., *traG*, *virB*4 and *virD*4) in addition to the superoxide dismutase *sodA* of *S. ixodetis* were overexpressed in the ovaries.

**Conclusion:**

The symbiont *S. ixodetis* is a new member of the bacterial community of *D. coccus* and *D. opuntiae*. The recovery of incomplete virulence factor-encoding genes in *S. ixodetis* of *Dactylopius* suggests that this bacterium is a non-pathogenic symbiont. A high number of genes encoding the T4SS, in the *S. ixodetis* genomes and the overexpression of these genes in the ovary and hemolymph of the host suggest that *S. ixodetis* use the T4SS to interact with the *Dactylopius* cells. Moreover, the transcriptional differences of *S. ixodetis* among the gut, hemolymph and ovary tissues of *D. coccus* indicate that this bacterium can respond and adapt to the different conditions (e.g., oxidative stress) present within the host. All this evidence proposes that there is a strong interaction and molecular signaling in the symbiosis between *S. ixodetis* and the carmine cochineal *Dactylopius*.

**Supplementary Information:**

The online version contains supplementary material available at 10.1186/s12864-021-07540-2.

## Background

Insects are associated with a plethora of microorganisms partitioned in different tissues of the host. Particularly, these bacterial symbionts can be distributed in the gut, hemolymph and even in the reproductive organs of the insect host [1]. An example of multi-tissue colonizer bacteria are some members of *Spiroplasma*.

The genus *Spiroplasma* comprises wall-less, motile, and helical bacteria of the class Mollicutes. These bacteria are mainly associated with insects but there are also reports of *Spiroplasma* in arachnids, crustaceans and plants [2, 3]. In insects, *Spiroplasma* can be vertically transmitted from females to offspring [4]. Transmission to plants involves an insect vector, as has been observed with the plant pathogen *S. citri* which is transferred to plants by leafhoppers and psyllids [5]. *Spiroplasma* associated with insects consist of mutualists, commensals, male-killing reproductive parasites and pathogens [6]. There are a few reports of pathogenic spiroplasmas in insects, like *S. apis* and *S. melliferum*, which produce lethal infections in honeybees [7, 8]. Nonetheless, most reported spiroplasmas are beneficial and may be considered facultative symbionts [3]. In addition to lethal pathogenicity, two other phenotypes are induced by spiroplasmas in insects such as protection against parasites (wasps and nematodes) and male-killing. Both phenotypes are produced by *Spiroplasma poulsonii* in *Drosophila* [9–11]. Protection against parasites has been associated with the presence of genes encoding ribosomal inactivating proteins (RIPs) in *S. poulsonii* genome. These RIPs are toxins that damage the ribosomes of *Drosophila* parasitic wasps and nematodes [12, 13]. Likewise, a plasmid-encoded protein (Spaid) seems to be involved in *D. melanogaster* male-killing phenotype produced by *S. poulsonii* [11, 14].

Molecular and phylogenetic classification using either the 16S rRNA or single-copy gene markers have split *Spiroplasma* spp. into four major clades. Three clades are composed of the formally described *Spiroplasma:* Citri-Chrysopicola-Mirum (CPM), Apis, and Ixodetis. The remaining clade (Mycoides-Entomoplasmataceae) contains species from the genera *Mycoplasma*, *Mesoplasma* and *Entomoplasma*, that have lost the helical cell morphology (thus they are not named *Spiroplasma*) [15]. *Spiroplasma* genome size ranges between 0.7 to 2.2 megabase pairs (Mbp). To date, 32 genomes of *Spiroplasma* spp. are available. However, only two belong to the Ixodetis clade, one is from the parasitic wasp *Cephus cinctus* [16] and the other is from the African monarch butterfly *Danaus chrysippus* [17], which makes this clade the least represented at the genomic level, in spite of being present in many insects [18].

Up to now, there are few functional genomic studies of *Spiroplasma*, two of them used quantitative reverse transcriptase PCR (qRT-PCR) to analyze the change of expression in key genes of *S. citri* [19, 20]. Additionally, there are two total transcriptional studies (RNAseq) of spiroplasmas, one analyzed the differences in culture growth of *S. diminutum* and *S. taiwanense* isolated from mosquitos [21], and other described the transcriptional profile of *S. poulsonii* in *Drosophila* (hemolymph) and in culture medium [22]. Differential gene expression was evidenced in all studies under contrasting conditions. Nonetheless, to the best of our knowledge, there is no transcriptome analysis of spiroplasmas colonizing different insect host tissues and the genes involved in the symbiotic interaction. Moreover, there is no transcriptional study of *S. ixodetis*.

*Dactylopius* (Hemiptera: Coccoidea: Dactylopiidae) is a scale insect that feeds on the sap of prickly pear, mainly from the genus *Opuntia* [23]. *Dactylopius* is the main source of carminic acid, a red dye used in cosmetics, drugs, food, and textile industry, achieving economical relevance [24]. The genus *Dactylopius* comprises more than 10 species, but only *D. coccus* is used for the extraction of carminic acid. This species was probably domesticated in Mexico more than one thousand years ago, selected for increased production and quality of the pigment compared to the other ‘wild’ species (e.g., *D. opuntiae*) [23, 25, 26]. Previous reports using metagenomic, metatranscriptomic and culture approaches showed diverse bacteria and fungi in both the domesticated and wild carmine cochineals [25, 27–30]. Within the microbial community present in *Dactylopius*, we reported a nitrogen-fixing β-proteobacterium *Candidatus* Dactylopiibacterium carminicum [30, 31] as well as two different strains of *Wolbachia* (*w*DacA and *w*DacB) [29], and fungi [32]. As part of the metagenome and metatranscriptome surveys from *D. coccus,* we also reported the presence of *Spiroplasma* sequences [31]. However, there is no study of the phylogenetic classification, genomic capabilities, and the putative roles of *Spiroplasma* bacterium within *Dactylopius*. Here we present the analysis of three *Spiroplasma ixodetis* metagenome-assembly genomes (MAGs) independently recovered from the domesticated *D. coccus* females and males, of two different populations each, as well as from a population of the wild cochineal *D. opuntiae* (females) metagenomes. Additionally, we used the previously reported metatranscriptome data from the gut, hemolymph, and ovary of *D. coccus* [31], to analyze the expression of genes putatively involved in the symbiotic interaction between *S. ixodetis* and *Dactylopius.* Genomic and metatranscriptomic analyses here presented, suggest that *S. ixodetis* may use the type IV secretion system (T4SS) to interact with the carmine cochineal. Moreover, the incompleteness of genes encoding virulence factors indicates that *S. ixodetis* could be considered as a non-pathogenic symbiont.

## Results and discussion

### New *Spiroplasma ixodetis* symbiont is present in multiple *Dactylopius* spp. metagenomic samples

**Fig. 1:**
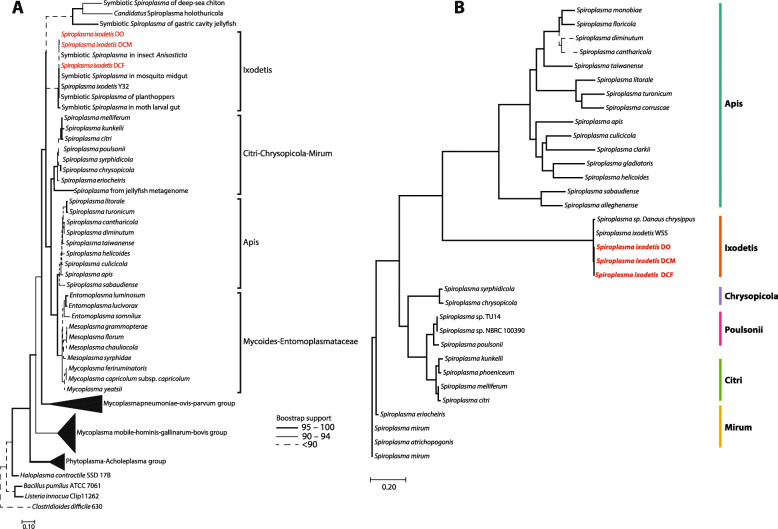
Phylogenetic position of *Spiroplasma* symbionts associated with *Dactylopius* cochineals. Maximum-likelihood trees of 16S rRNA genes (a) and 168 concatenated single-copy gene markers (b). Scale bars indicate 10 and 20% estimated sequence divergence, respectively. See Additional file 2 Data set 1 for the accession numbers of the sequences used in these analyses

Mollicute-related MAGs were recovered in metagenomic assembled and binned samples from adult males and females of the domesticated carmine cochineal *D*. *coccus* from two different populations, as well as in the metagenome from adult females of the wild cochineal species *D. opuntiae*. Analysis of single-copy gene markers showed that these MAGs exhibited high completeness (> 92%) and no apparent contamination (Table 1). Mollicute MAGs of *D. opuntiae* females*, D. coccus* females and males were placed into 258, 286 and 353 scaffolds, respectively (Table 1). The estimated genome size of these MAGs ranged from 1.32 to 1.9 Mbp. Around 1215 to 1371 coding sequences (CDS) were identified within these genomes (Table 1). Like in other Mollicutes [33], the Mollicutes-related MAGs from the *Dactylopius* spp. had low G-C % content (~ 24%, Table 1). The complete sequences of the 16S rRNA gene were obtained for all three MAGs (Table 1). Phylogenetic reconstruction of these 16S rRNA sequences placed the *Dactylopius* Mollicute-related MAGs within the *Spiroplasma* genus in the Ixodetis clade. (Fig. 1a). Additionally, 16S rRNA sequence from *D. coccus* and *D. opuntiae* MAGs showed 99% nucleotide identity with the *S. ixodetis* Y32 16S rRNA sequence from the western black-legged ticks (*Ixodes pacificus*) and to other *S. ixodetis* (Additional file 1 Fig. S1). We further refer to *Dactylopius* associated *Spiroplasma* as *S. ixodetis* DO (from *D. opuntiae*, female), *S. ixodetis* DCF (from *D.coccus*, female) and *S. ixodetis* DCM (from *D. coccus* male), respectively.

**Table 1:** General genomic features of *Spiroplasma ixodetis* DO, DCF and DCM compared to othersinsect-associated *Spiroplasma*

Genome ID	***S. ixodetis*** DO	***S. ixodetis*** DCF	***S. ixodetis*** DCM	S. *ixodetis* WSS	*Spiroplasma* sp.	*S. melliferum*	*S. sabaudiense*
**Number of scaffolds/contigs**	258	286	353	145	12	6	1
**Estimated genome size (Mb)**	1.28	1.19	1.32	0.73	1.75	1.29	1.075
**Average genomic coverage**	1795x	1480x	1112x	0.727x	2000x	11x	600.8x
**N50**	8196	6014	7774	5160	265,779	741,187	NA
**G + C content (%)**	24.63	24.21	24.16	24.56	23.7	27.09	30.16
**CDS genes**	1260	1215	1371	649	1813	1297	933
**rRNA (16S, 5S, 23S)**	2(1,1,ND)	2(1,1,ND)	3(1,1,1)	NA	4(1,2,1)	3(1,1,1)	6(2,2,2)
**tRNA**	27	27	27	23	27	31	32
**Genome completeness (%)**^a^	95.5	92.5	94.7	77.8	95.5	98.1	100
**Insect associated**	*Dactylopius opuntiae*	*Dactylopius coccus*		*Cephus cinctus*	*Danaus chrysippus*	*Apis mellifera*	*Aedes* sp.

The number of CDS genes, rRNA, tRNA for *Dactylopius* associated *S. ixodetis* was calculated using the Prokka annotation files. Otherwise, information was retrieved from the NCBI GenBank database

^a^Genome completeness was calculated for all organisms with the checkM pipeline

NA not available, ND not detected

To further classify and compare the *S. ixodetis* from *Dactylopius* cochineals with other spiroplasmas, we performed a pan-genome analysis using 30 public *Spiroplasma* genomes from the Apis, Ixodetis, Chrysopicola, Poulsonii, Citri and Mirum phylogenetic clades (Additional file 2 Data set 1). The pan-genomic analysis showed 11,045 gene clusters and 168 of them corresponded to single copy core-genes within the *Spiroplasma* pan-genome. Robust phylogenomic analysis using the 168 single-copy core genes confirmed the position of spiroplasmas from *Dactylopius* within the Ixodetis clade (Fig. 1b). Average amino acid identity (AAI), using the same 168 core single-copy genes, showed that *S. ixodetis* DO, DCM and DCF shared identities of ~ 99.5% with the genomes of *Spiroplasma ixodetis* symbiont of the wheat stem sawfly (WSS) *Cephus cinctus* [16] and ~ 98.5 with the *Spiroplasma* endosymbiont of the African monarch butterfly *Danaus chrysippus* [17] (Additional file 2 Data set 1). AAI analysis also showed that *S. ixodetis* in both female and male *D. coccus* populations were 100% identical (Additional file 2 Data set 1). Moreover, *S. ixodetis* of *D. opuntia* showed a 99.78% AAI value in comparison to *S. ixodetis* of both *D. coccus* populations (Additional file 2 Data set 1). This result suggests that there are slight variations in the bacterial genomes between the two *Dactylopius* species (i.e., *D. coccus* and *D. opuntiae*), although there is no apparent difference between spiroplasmas of the two populations (males and females) of *D. coccus*. Similar variations in core gene identity (ANI ~ 99.5) have been reported between the sister species strains of *S. poulsonii* (*s*Mel and *s*Hy), symbionts of *Drosophila melanogaster* and *D. hydei*, respectively, and it has been linked to a host adaptation process [34]. Likewise, genomic differences between the *S. ixodetis* strains of *Dactylopius* may result from adaptation to different host species (i.e., *D. opuntiae* and *D. coccus*).

To find out if *S. ixodetis* was present in other *Dactylopius*, we further analyzed the metagenomes of *D. coccus* (females), from commercial samples of Mexico and Peru, previously reported by Campana et al., [35]. No complete *S. ixodetis* MAGs were recovered after assembly and binning analyses of these *D. coccus* metagenomes. Nonetheless, we were able to identify long (> 900 base-pairs [bp]) and highly similar (BLASTn identity > 90% and e-value < 0.0005) contigs to those from the *S. ixodetis* DCF genome in both the Mexican (*n* = 56) and Peruvian (*n* = 20) metagenomic assemblies. The above confirms that *S. ixodetis* is distributed not only in different species of *Dactylopius*, but also in different populations of *D. coccus*. This indicates that this bacterium can form a seemingly symbiotic relationship with the carmine cochineal and may not be a merely sporadic association. As in nature, frequencies of *Spiroplasma* spp. in their insect host are variable [36, 37], further studies are required to elucidate the prevalence of *S. ixodetis* in other *Dactylopius* species/populations.

### Reduced number of genes for amino acid and carbon metabolism were found in *S. ixodetis* DO, DCM and DCF

**Fig. 2:**
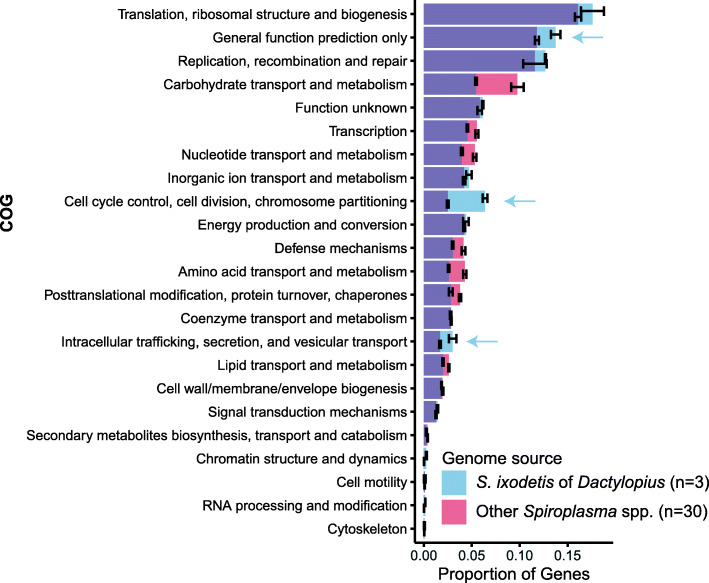
Comparative genomics of *Spiroplasma* associated with *Dactylopius* and other *Spiroplasma*. COG profiles of *S. ixodetis* DCF, DCM and DO (blue bars) compared to other *Spiroplasma* genomes (red bars). Blue arrows indicate enrichment of genes in the COG categories on *S. ixodetis* DCF, DCM and DO genomes. The mean ± SEM proportion of genes belonging to each COG category is shown

To gain information about the general metabolic profiles of *S. ixodetis* and their role in the interaction with *Dactylopius*, a comprehensive comparative genomic analysis was performed between *S. ixodetis* DO, DCM and DCF and other *Spiroplasma* (*n* = 30) genomes recovered from diverse environments (i.e., vertebrates, plants, and other insects). Analysis of the clusters of orthologous groups of proteins (COG) showed that *S. ixodetis* DO, DCM and DCF have fewer genes associated with transport and metabolism of amino acids compared to other *Spiroplasma* spp. genomes (Fig. 2). *Spiroplasma* species are auxotrophs for most of the essential amino acids and require multiple transporters to obtain them from the host [38]. A smaller number of genes coding for ABC transporters of peptides and oligopeptides as well as fewer genes involved in the biosynthesis of amino acids were detected in *S*. *ixodetis* genomes from *Dactylopius* in comparison with other *Spiroplasma* spp. (Fig. 2 and Additional file 3 Data set 2). Most of the *Spiroplasma* obtain ATP through arginine metabolism [39, 40]. However, and contrasting with the previous *S. ixodetis* WSS genome [16], no arginine biosynthetic genes were found in *S. ixodetis* of *Dactylopius* (Additional file 3 Data set 2).

A complete set of genes for glycolysis, fructose catabolism and the pentose-phosphate pathway were found in *S. ixodetis* DO, DCM and DCF genomes (Additional file 3 Data set 2). Trehalose, glucose and mannose are abundant sugars in insect hemolymph and most of the insect-associated spiroplasmas encode genes involved in the phosphotransferase system (PTS) to transport these sugars into the bacterial cell [38, 40, 41]. Accordingly, *S. ixodetis* DO, DCM and DCF showed genes encoding glucose (*ptsG*), fructose (*fruA/B*) and N-acetyl-glucosamine (*nagE*) PTS transporters (Additional file 3 Data set 2). Even though *S. ixodetis* DO, DCM and DCF display genes for maltose and cellobiose PTS systems, no other genes for oligosaccharides catabolism were found in these genomes (Additional file 3 Data set 2). Particularly, no genes coding for oligosaccharide breakdown and catabolism (e.g., glycoside hydrolases) were found in DO, DCM and DCF genomes (Additional file 3 Data set 2) suggesting that *S. ixodetis* is unable to utilize complex polysaccharides as a carbon source.

In *Spiroplasma* species that are insect pathogens, such as *S. citri, S. apis* and *S. mellipherum*, genes for trehalose utilization, including the PTS, are present [40–42]. However, similar to *S. ixodetis* of *Dactylopius*, genes for trehalose catabolism are absent or found as non-functional pseudogenes in the non-pathogenic species *S. poulsonii*, *S. chrysopicola* and *S. syrphidicola* [40, 43]. The putative inability to metabolize trehalose by *S. ixodetis* DO, DCF and DCM may limit spiroplasma growth in *Dactylopius* tissues as has been previously suggested in the *Drosophila*-*S. poulsonii* interaction [40].

### *S. ixodetis* DO, DCM and DCF genomes have few virulence factors-encoding genes

Incomplete genes encoding toxin-like proteins and other putative virulence factors were found in the *S. ixodetis* DO, DCM and DCF genomes (Table 2). Ankyrin repeat domains are present in many virulence effector proteins [44]. Particularly, the ankyrin-repeat containing protein Spaid of *S. poulsonii* (locus SMSRO_SFP00290) contributes to the male-killing phenotype in *D. melanogaster* [11]. Incomplete homologs (> 50% identity, > 75% BLASTp coverage) of *spaid* gene were found in DO, DCM and DCF genomes (Table 2). Similarly, *spaid* homologs were reported in *S. ixodetis* WSS genome [16]. However, *spaid* sequences of *S. ixodetis* WSS lack the N-terminal signal peptide domains present in *S. poulsonii* Spaid protein and thus were classified as not functional proteins [16]. In addition to *spaid*, partial coding sequence, homologs to the ribosome-inactivating protein (RIP2) gene of *S. poulsonii* were found in *S. ixodetis* from the domesticated *D. coccus* but not from the wild species *D. opuntiae* (Table 2). RIP proteins of other spiroplasmas have been implicated in protection against nematodes and parasitic wasp in different species of *Drosophila* [45, 46]. Even though genomic results suggest *S. ixodetis* DO, DCM and DCF encode multiple toxin-like factors, most of these are incomplete or annotated as putative pseudogenes.

**Table 2:** Virulence factor-encoding genes present in *S. poulsonii* and homologous genes in *Dactylopius*-associated *S. ixodetis*

			*S. ixodetis*		
	Annotation in *S. poulsonii* ^a^	*S. poulsonii* locus id ^a^	DCF locus id	DCM locus id	DO locus id
Toxins	Ankyrin repeat (Spaid)	SMSRO_SFP00290	DC_DC_00469^b^	KIIIGDCO_00556^b^	DO_DO_00453^b^
	ETX-like	SMSRO_SF021610	–	–	–
	RIP1	SMSRO_SF016530	–	–	–
	RIP2	SMSRO_SF018820	DC_DC_00311^b^	KIIIGDCO_00357^b^	–
	RIP3	SMSRO_SF023880	–	–	–
	RIP4	SMSRO_SF020720	–	–	–
	RIP5	SMSRO_SF003660	–	–	–
Adhesins	SpARP1^d^	SMSRO_SF002520	–	–	DO_DO_00287
	SpARP2	SMSRO_SF011850	–	–	–
	SpARP3	SMSRO_SF022680	–	–	DO_DO_00279
	SpARP4	SMSRO_SF024450	–	KIIIGDCO_00941	–
	SpARP5	SMSRO_SFP00390	–	–	–
Spiralins	SpiA	SMSRO_SF013140	–	–	–
	SpiB	SMSRO_SF009660	–	–	–
	SpiC	SMSRO_SF015890	–	–	–
Chitinases	ChiD1	SMSRO_SF008450	–	–	–
	ChiD2	SMSRO_SF013110	–	–	–
Lipid metabolism	Cls	SMSRO_SF001010	DC_DC_00075	KIIIGDCO_00120	DO_DO_00089
	GlpO	SMSRO_SF018440	DC_DC_00364^c^ DC_DC_00365^c^	KIIIGDCO_00489^c^ KIIIGDCO_00490^c^	DO_DO_00848^c^ DO_DO_00849^c^

^a^Masson et al., 2018

^b^ Incomplete or partial protein

^c^ Pseudogene

^d^ Annotated as Putative adhesin P89 in *S. citri* (Uniprot ID: Q9EV58_SPICI)

In *S. poulsonii* and *S. citri* spiralin protein, encoded by *spiA*, *spiB* and *spiC*, acts as a lectin anchor for binding to glycoproteins present in the insect cells and is required for efficient colonization of the host [39, 47, 48]. No homologous gene of spiralin *spiA*, *B* or *C* was detected in *S. ixodetis* DO, DCM and DCF genomes (Additional file 3 Data set 2). Nonetheless, homologous genes encoding the adhesins SARP1 and SARP3 from *S. poulsonii* and *S. citri* were found in *S. ixodetis* DO and DCM genomes (Table 2). Adhesins like SARP1 and SARP3 as well as phosphoglycerate kinase (PGK) are involved in cell adherence and invasion of *S. citri* to the leafhoppers *Circulifer tenellus* and *Circulifer haematoceps* [49, 50]. All three *S. ixodetis* DO, DCM and DCF genomes encode PGK (Additional file 3 Data set 2) which in *S. citri* is a key factor for bacterial internalization into the host cells [51, 52].

**Fig. 3:**
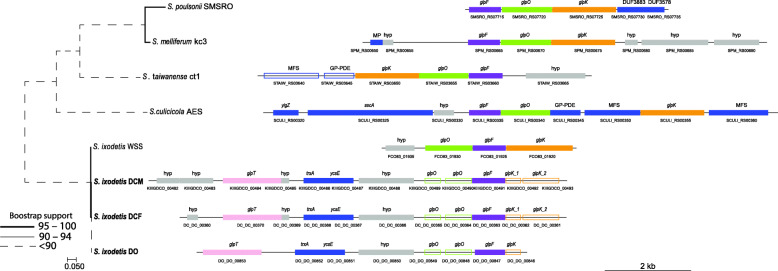
Gene structure of the glycerol catabolic genes (*glpF*, *glpO* and *glpK*) present in different *Spiroplasma* spp. genomes. Color-empty blocks represent pseudogenes. Gray blocks show genes annotated as hypothetical (hyp) protein. Maximum-likelihood phylogenetic tree in the left shows the cladogenesis of *Spiroplasma* spp. genomes using the Glycerol-3-phosphate oxidase encoding gene *glpO*. MAFFT was used to align all *glpO* sequences from each genome and the phylogenetic tree, based on the LG + G4 substitution model obtained by ModelFinder, was calculated by IQtree with 1000 Bootstrap replicates for internal branch support. Scale bar indicates 5% estimated sequence divergence

Glycerol catabolic and transporter genes were found in *S. ixodetis* DO, DCM and DCF genomes (Additional file 3 Data set 2), particularly, the genes *glpO* in addition to *glpF* (membrane channel) and *glpK* (kinase) (Fig. 3). Synteny analysis showed similar genomic architecture of *glpO*, *glpF* and *glpK* genes in *S. ixodetis* DO, DCM and DCF genomes and in *S. poulsonii¸ S. melliferum* and *S. culicicola* (Fig. 3), which agrees with previous reports of *glp* gene organization in other spiroplasmas [53]. Glycerol metabolism may lead to the production of hydrogen peroxide (H_2_O_2_) by L-α-glycerophosphate oxidase (GlpO) [3, 54]. In the human pathogen, *Mycoplasma mycoides* subsp. *mycoides*, H_2_O_2_ has been linked to tissue inflammation and cellular damage [55, 56]. Thus, in insect associated pathogenic *Spiroplasma* spp. (i.e., *S. taiwanense*, *S. culicicola* and *S. apis*) the presence of *glpO* coding gene has been considered to be a virulence factor [57]. However, unlike other spiroplasmas, *glpO* and *glpK* genes in *S. ixodetis* DO, DCM, and DCF were incomplete and annotated as pseudogenes suggesting that they are unable to produce H_2_O_2_ from glycerol (Fig. 3).

*glpO*, *glpF* and *glpK* genes are commonly recovered from pathogenic *Spiroplasma* like *S. culicicola* and *S. taiwanense*. In those bacteria n-glycerol 3-phosphate (G3P) is taken up by the transporters UgpA, UgpC and UgpE [57]. Even though no *ugpA/C/E* homologous genes were found in *S. ixodetis* genomes (Additional file 3 Data set 2), a *glpT* gene, encoding the G3P-transporter, was found in the DO, DCM and DCF genomes, but not present in other *Spiroplasma* genomes of insects (Fig. 3). Comparative analysis showed that this gene forms an orthologous cluster with the *glpT* of *S. floricola*, *Mycoplasma yeatsii* and *Mycoplasma putrefaciens*. This suggests that in *S. ixodetis* the G3P might be transported using a GlpT transporter instead of the UgpA/C/E system which is associated with pathogenicity.

Altogether, the absence of genes for trehalose catabolism, the pseudogenization of virulence factors (i.e., *spaid* and *glp*O) as well as the presence of genes for insect cell colonization (i.e., the adhesins SARP1, SARP3 and PKG), suggest *S. ixodetis* is adapted to live as a non-pathogenic symbiont inside *Dactylopius*.

### Comparative genomics revealed an enrichment of genes encoding the type IV secretion system (T4SS) in the *Dactylopius* associated S*. ixodetis* in comparison to other *Spiroplasma* spp. genomes

**Fig. 4:**
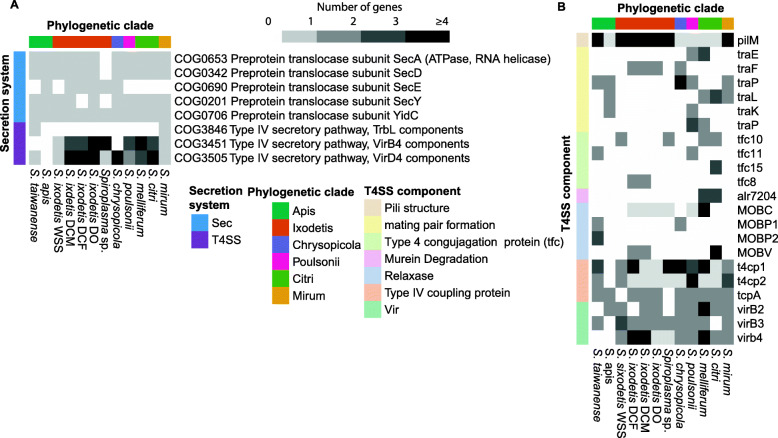
Differences in genes encoding secretion systems among *Spiroplasma* spp. genomes. Heatmaps showing the number of genes associated with (a) Intracellular trafficking, secretion, and vesicular transport COG category. (b) Heatmap showing the number of genes associated with the type IV secretion system (T4SS) among all the *Spiroplasma* spp. genomes. Colors at the top of the heatmaps indicate the *Spiroplasma* phylogenetic position of each genome

COG profile comparison between DO, DCM and DCF and other *Spiroplasma* spp. revealed a greater representation of the intracellular trafficking/secretion category in DO, DCM and DCF (Fig. 2). Secretion of macromolecular effectors (i.e., protein and nucleic acids) plays a central role in modulating the interactions between symbiotic (pathogenic and mutualistic) bacteria and their host [58, 59]. Similar to other spiroplasmas associated with insects (e.g., *S. poulsonii*) [40], *S. ixodetis* DO, DCM and DCF showed genes encoding the general secretion (Sec) system. Particularly, the genes *secA*, *secY* (missing in DCF), *secG* and *secE* were found in *S. ixodetis* from *Dactylopius* spp. (Fig. 4a). Other Sec coding genes such as the signal recognition GTPase (*ffh* and *ftsY*) and the translocase (*yidC*) were also present in *S. ixodetis* DO, DCM and DCF genomes (Additional file 3 Data set 2) suggesting DO, DCM and DCF use the Sec system to export proteins.

Additionally, *S. ixodetis* DO, DCM and DCF genomes showed more *virB4*-*D4* predicted genes, associated with the type IV secretion system (T4SS), than other *Spiroplasma* species associated with insects (Fig. 4a). A phylogeny using the VirB4 ATPase clustered the *virB4* sequences of *S. ixodetis* DO, DCM, and DCF with other *virB4* of *Spiroplasma* spp. (Additional file 1 Fig. S2), discarding a putative chimeric-assembly origin of these genes. Additionally, multiple *virB4-D4* genes were found in scaffolds encoding plasmid-like coding sequences (i.e., *soj* and plasmid replication protein; Additional file 1 Fig. S3 and Additional file 3 Data set S2) in the *S. ixodetis* DO genome. Differences in coverage of genomic contigs have been used as a proxy to detect plasmids in bacteria [60] and plasmid-like scaffolds with *virB4-D4* sequences of *S. ixodetis* DO showed a higher sequence coverage (> ~ 5000x) than the coverage for other scaffolds in the DO genome (Additional file 1 Fig. S3). Similarly, genes encoding VirB4 T4SS ATPase were detected in plasmids of *S. kunkelii* and *S. citri*, pSKU146 and pSci1–6, respectively [61, 62]. A maximum-likelihood phylogenetic analysis showed that the sequence of *S. ixodetis* DO *virB4* (DO_DO_00343) cluster together with the *virB4* genes from plasmids pSKU146 and pSci1–6 (Additional file 1 Fig. S4). All this evidence suggests that like in *S. citri* and *S. kunkelii* the T4SS of *S. ixodetis* DO is plasmid borne. Although the DCM and DCF genomes have copies of genes *soj*/*parA*, *smc*/*spo0J* and the replication protein COG5521, involved in plasmid segregation [63–65], no *virB4*-*D4* genes were found in the plasmid-like scaffolds of these bacteria (Additional file 3 Data set 2).

Annotation using the TXSSdb database (see methods) revealed that other T4SS elements are present in *S. ixodetis* DO, DCM and DCF genomes such as genes coding for the type IV coupling protein (t4cp), the MOB relaxase and *tra* genes (Fig. 4b). These elements are typically used by bacteria in the conjugation process [66]. Previously, the presence of *mob* and *tra* coding genes in *S. citri* plasmid pSKU146 suggested that spiroplasmas are conjugative [62]. Having similar elements in DO, DCM and DCF indicates that these bacteria might be conjugative. Not all the components for the T4SS were found in *S. ixodetis* DO, DCM and DCF genomes (Fig. 4a-b), however, it has been suggested that Gram-positive bacteria, and specifically the cell wall lacking members of the *Mollicutes*, do not require as many T4SS components to secrete macromolecules as the Gram-negative bacteria [61]. Thus, *S. ixodetis* DO, DCM and DCF may have a functional T4SS. In *Wolbachia*, perhaps the most abundant insect intracellular-symbiont, multiple T4SS loci encoding Vir proteins were expressed during all stages of the host development from embryogenesis to adult male and female *Drosophila* flies [59]. Moreover, it has been considered that *Wolbachia* uses the T4SS to secret as many as 105 proteins with putative effector function to the host (*Drosophila*) cells [53]. Like *Wolbachia* and other symbiotic bacteria (e.g., *Mesorhizobium loti*) [67], T4SS could be used by *S. ixodetis* to interact with the host.

### *S. ixodetis* DCF differential gene expression in the gut, hemolymph, and ovary tissues of *D. coccus*

**Fig. 5:**
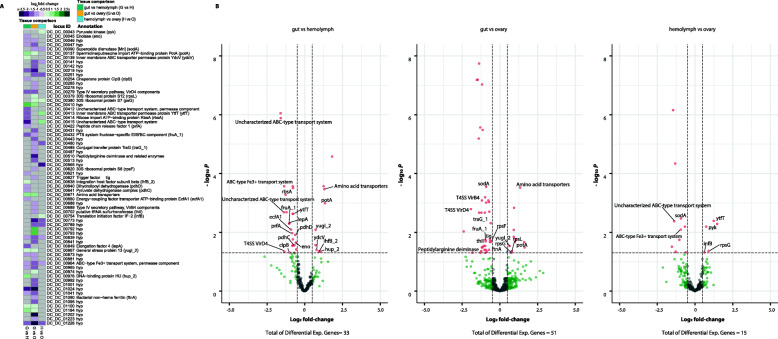
Variation on gene expression of *S. ixodetis* DCF in the gut, ovary, and hemolymph of *D. coccus*. (a) Heatmap showing all the differentially expressed genes in each tissue comparison (i.e. gut vs hemolymph, gut vs ovary and hemolymph vs ovary). Color range in heatmaps indicates variation in log2 fold-change. Gray boxes indicate no differential expression observed after DESeq2 analysis (absolute log_2_ fold-change ≥0.58 and *p*-adjust value ≤0.05). hyp means genes annotated as hypothetical proteins. (b) Volcano plot of differential gene expression of *S. ixodetis* DCF in gut vs hemolymph, gut vs ovary and hemolymph vs ovary comparisons. Red dots show genes considered as differentially expressed after DESeq2 analysis absolute log_2_fold change > 0.58 (outer broken vertical lines) and *p*-adjust value ≤0.05 (−log_10_ *p*-value ≥1.3, broken horizontal line). Green dots show non-differentially expressed genes with an absolute log_2_ fold-change > 0.58 and *p*-adjust value > 0.05. Gray dots show non-differentially expressed genes with an absolute log_2_fold change < 0.58 and *p*-adjust value > 0.05 (non-differentially expressed). All annotated genes are represented, red dots without annotation legend represent hypothetical protein coding genes differentially expressed

To detect genes seemingly involved in the interaction between *S. ixodetis* and *Dactylopius*, we compared the transcriptome of *S. ixodetis* DCF recovered from previously reported metatranscriptomic data of *D. coccus* gut, hemolymph, and ovaries [31]. After ribosomal sequences depletion, *ca*. 1 million metatranscriptomic reads mapped to *S. ixodetis* DCF genome in each of the *D. coccus* gut, hemolymph, and ovary metatranscriptomic samples (Additional file 1 Fig. S5a). Principal component analysis (PCA) of *S. ixodetis* DCF transcripts showed that the transcriptomes differed along with the ordination space by intrasample variation (y-axis 21% of variance) and tissue specificity (i.e., gut, hemolymph, and ovary; x-axis 30% of variance; Additional file 1 Fig. S5b). The above suggests that *S. ixodetis* DCF modulate gene expression depending on the allocation in the different host tissues. Pairwise comparisons between each tissue combination (i.e., gut vs. hemolymph, gut vs. ovary and hemolymph vs. ovary) identified 69 unique differentially expressed genes of *S. ixodetis* DCF across all comparisons and 46% matched with a coding product in the bacterial genomic annotation (Fig. 5a and Additional file 3 Data set 2). Among these genes, we detected that the conjugal transfer protein coding gene *traG*, as well as the *virB4* and *virD4* genes of the T4SS of *S. ixodetis* DCF, were up-regulated in the hemolymph and the ovaries in comparison to the gut (Fig. 5b). Additionally, other *S. ixodetis* DCF T4SS related encoding genes such as the type IV pilus inner membrane component *pilM*, the type four conjugation *tfc*, the relaxase *mob* and the *virB3* were also expressed, but not differentially (*p*-value > 0.05), in the *D*. *coccus* gut, hemolymph, and ovary (Additional file 3 Data set 2). Expression of *vir* genes, particularly *virB4*, encoding for the T4SS of *Wolbachia* has been previously detected in the ovaries of the wasp *Asobara tabida* suggesting that T4SS is active and could participate in the *Wolbachia*-*A. tabida* interaction. It has been suggested that *Wolbachia* might use T4SS to export effectors for manipulating the wasp cell biology and oogenesis [59, 68]. Similarly, high expression of *virB4* and *virD4* genes in *D. coccus* ovary suggests that the T4SS of *S. ixodetis* DCF is biologically active. An elevated number of T4SS coding genes in the *S. ixodetis* DCF genome and the up-regulation of T4SS genes in the hemolymph and ovaries of *D. coccus* (Fig. 5a-b and Additional file 3 Data set 2), indicate that the T4SS might play an important role in the interaction between *S. ixodetis* DCF and the host.

Metatranscriptome analysis also showed that *S. ixodetis* DCF genes encoding the Spaid and GlpO proteins, described as virulence factors*,* have low average levels of expression (≤ 42 fragments per kilobase of transcript per million mapped reads [FPKM]) and were not differentially expressed in any of the *D. coccus* tissues analyzed. Moreover, no expression of *S. ixodetis* RIP encoding gene was detected in any of the *D. coccus* metatranscriptomes (Additional file 3 Data set 2). In *S. poulsonii*, genes encoding the RIP and GlpO proteins were up-regulated in *D. melanogaster* hemolymph in contrast to in-vitro cultures, indicating an important role of these genes in the fly-*S. poulsonii* interaction [22]. The low expression values of these spiroplasma genes in all the *Dactylopius* tissues, as well as the fact that these genes are incomplete, suggests that genes encoding virulence factors might not have an important role for the *D. coccus*-*S. ixodetis* interaction.

A high expression of *S. ixodetis* DCF genes associated with reactive oxygen species (ROS) catabolism such as the *sodA*, encoding the superoxide dismutase, was observed in the *D. coccus* ovaries relative to the gut and hemolymph. Additionally, genes *pdhC* and *pdhD*, encoding the dihydrolipoyl dehydrogenase, were up-regulated in the hemolymph relative to the gut (Fig. 5a-b and Additional file 3 Data set 2). In the intracellular bacteria *Mycobacterium tuberculosis* the expression of *pdhC* and *pdhD* has been associated with resistance to host-reactive nitrogen intermediates [69]. ROS and reactive nitrogen species (RONS) play a critical role in the establishing, maintenance, and success of the symbiosis [70]. As other eukaryotes, insects control bacterial symbionts load in their tissues by producing RONS. Nonetheless, some bacteria, mainly mutualistic bacteria, can cope with the burst of host RONS by displaying an arsenal of antioxidant enzymes (e.g., superoxide-dismutase and peroxidase) [70]. For example, in the mutualistic association between the bobtail squid *Euprymna scolopes* and the luminous bacteria, *Vibrio fisheri*, bacteria can overcome the squid RONS by over expressing a peculiar NO/oxygen-binding (H-NOX) protein to catabolize the nitric oxide produced by the host. This allows *V. fisheri* to successfully colonize the highly oxidative light-organ tissue of *E. scolopes* [71]. Likewise, *Wolbachia*, possess a single *sod* gene that likely plays a fundamental role in the management of oxidative stress produced by insects’ cells [72]. In many insects, *Spiroplasma* reside as extracellular bacteria in the gut and the hemolymph, however, in other tissues such as the fat body and ovaries these bacteria are found as intracellular symbionts [4, 38, 73]. The up-regulation of *S. ixodetis* DCF *sod* gene in the ovaries compared to the gut indicates that like *Wolbachia*, *Spiroplasma* could use the superoxide-dismutase to manage the oxidative stress produced inside the insect ovarian cells. This strategy might allow *S. ixodetis* DCF to colonize the embryonic cells and be vertically transmitted to the *Dactylopius* offspring.

## Conclusions

Microbial associations with insects are considered key to their biological success. In many cases, there are multiple microbes within a single insect, and that is the case of *Dactylopius* spp. which is associated with the diazotroph bacterium *Candidatus* Dactylopiibacterium carminicum and two *Wolbachia* strains. Here, we described that *Spiroplasma ixodetis* is also a member of the bacterial community of both the wild (*D. opuntiae*) and the domesticated (*D. coccus*) carmine cochineals. The presence of *S. ixodetis* in three independent metagenomic samples of different populations of *Dactylopius* from two species (*D. coccus* and *D. opuntiae)* as well as in the metagenomes of other two different *D. coccus* metagenomes from Peru and Mexico, indicates that *S. ixodetis* is commonly found within *Dactylopius*. However, since these metagenome studies were performed with pools of insects, we cannot conclude that each individual cochineal has *Spiroplasma*. In *Drosophila* not all individuals of same population are infected with *Spiroplasma* and the same phenomenon should be further explored in the carmine cochineals. The genome analysis showed that females and males from different populations of the domesticated cochineals carry the same *S. ixodetis*, with slight differences from that of the wild cochineals, suggesting a common origin of the symbiosis.

The *Dactylopius* associated *S. ixodetis* genomes showed pseudogenization or incompleteness of genes commonly coding for virulence factors in other *Spiroplasma* spp. As no complete virulence genes were detected, *S. ixodetis* in *Dactylopius* could be considered a non-pathogenic symbiont. Transcriptomic analyses of endosymbionts in insects are scarce. Here the transcriptomic analysis of *S. ixodetis* DCF showed that genes involved in the T4SS (e.g., *traG*, *virB4* and *virD4*) were over expressed in hemolymph and ovary of the host *D. coccus*. Moreover, genes involved in oxygen-stress tolerance such as *sodA* were also over expressed in the ovary. These results suggest that a) *S. ixodetis* might use T4SS to interact with *Dactylopius* cells, and b) these bacteria can tolerate oxygen-stress produced inside the host cells. Finally, transcriptional differences of *S. ixodetis* among *Dactylopius* tissues suggest that this bacterium can respond and adapt to the different conditions present within the host tissues indicating that there is a strong interaction and molecular signaling in the symbiosis between *S. ixodetis* and the carmine cochineal *Dactylopius*.

## Methods

### Insect sampling

Females of *Dactylopius coccus* were obtained from Campo Carmín Company, Morelos, Mexico (18°44′46.7″N, 99°11′17.8″W) in April 2012. Males from a different population of *D. coccus* were obtained from a commercial farm in Tepoztlán, Morelos, Mexico (18°59′24.8″N 99°07′02.9″W) in February 2017. Females of wild cochineal *D. opuntiae* were collected from a cactus farm field in Morelos, Mexico (18°59′25.3″N 99°07′02.6″W) in May 2017. Insects were obtained from *Opuntia* spp. cactus and were transported together with their host plants to the laboratory for further experiments.

### DNA extraction and metagenomic sequencing

Shotgun metagenomes were previously reported and obtained [29, 32] using different sequencing technologies (i.e., 454-GS-FLx, Illumina HiSeq2000–2500 and PacBio) of total DNA from guts, Malpighian tubules, ovaries and hemolymph of *Dactylopius coccus* females. Details of the insect dissection and DNA extraction procedures from different *Dactylopius* tissues are fully described in [29, 30, 32]. Briefly, for 454 sequencing, 20 *D. coccus* adult females were superficially disinfected with 70% ethanol, rinsed with sterile distilled water, and dissected with sterile forceps to remove the exoskeleton and guts. Cells in the hemolymph and debris were separated by centrifugation in a Percoll gradient as is described by [74]. Percoll phases were observed under a microscope, and those with cells were selected for DNA extraction. For Illumina HiSeq2000 sequencing guts, ovaries, and Malpighian tubules from 40 females were dissected using sterile forceps under a stereoscopic microscope. We tried to avoid tissue cross-contamination by rinsing the tissues several times with sterile phosphate-buffered saline to remove remnant hemolymph. These organs were pooled, suspended in sterile PBS, and macerated using a sterile plastic pestle. For PacBio sequencing, eight individuals were superficially disinfected with 70% ethanol as above. Guts and exoskeleton were removed with sterile forceps and hemolymph from all individuals was pooled for DNA extraction. In addition, metagenome sequences of wild *Dactylopius opuntiae* females and males of different *D. coccus* population were obtained by Illumina HiSeq2500 platform. For these last metagenomes, two independent pools containing the whole body of 10 *D. opuntiae* females and 30 *D. coccus* males were superficially disinfected as described above. Insects were macerated using sterile pestles and resuspended in sterile PBS. Independently of the sequence technology, DNA from all insects was extracted with the DNeasy Blood & Tissue Kit (QIAGEN) following the manufacturer’s instructions. Sequencing was performed at Macrogen Inc. (Korea) for 454 and Illumina HiSeq2000–2500. PacBio sequencing was performed at Duke University Genome Sequencing Core Facility (USA). In all cases, after base calling high-quality metagenomic reads (quality Phred score > Q30) were obtained by adaptor removal and trimming using the TrimmGalore 0.4.4 pipeline (http://www.bioinformatics.babraham.ac.uk/projects/trim_galore/) with the --paired --q 30 options.

### Binning, genome assembly and annotation

For genomic binning all metagenomic reads from *D. coccus* females (i.e., 454, Illumina and PacBio), *D. coccus* males and *D. opuntiae* females were independently mapped using Bowtie2 2.3.5 [75] versus a concatenated index of *Wolbachia w*DacA (LSYX00000000.1), *Wolbachia w*DacB (LSYY00000000.1) and *Candidatus* Dactylopiibacterium carminicum (NZ_MQNN00000000.1) genomes, previously reported bacteria present in *Dactylopius* metagenomes [29, 32], with the --end-to-end and --very-sensitive options. All non-mapped reads from each metagenome (*D. coccus* female, *D. coccus* male and *D. opuntiae*, respectively) were retrieved from the bam files using Samtools 1.7 [76] with the following options: samtools fastq -@ 20 -f 2. To maintain read parity after samtools procedure, all fastq files from each metagenome were analyzed by TrimmGalore 0.4.4 pipeline with the --paired option, this resulted in high-quality paired reads for all libraries. These reads, from each *Dactylopius* species and populations, were then independently assembled using idba-ud 11.1 [77] with the default parameters. The resulting assembled genomic contigs were binned into single metagenomic assembled genomes (MAGs) by MaxBin 2.2.1 [78] with the following parameter -min_contig_length 900 for minimum contig length. In each binning the metagenomic contigs were divided into two different MAGs and classified by checkM 1.1.2 pipeline [79] with the lineage_wf option for a broad taxonomical classification. In the three metagenomes analyzed, checkM detected the presence of a Mollicute related MAG in addition to an insect-related MAG. To improve the assembly, the original high-quality reads were mapped to the MAG classified as Mollicutes lineage in each metagenome using Bowtie2 with the --end-to-end and --very-sensitive options. All paired and mapped reads were once again retrieved using samtools as above and reassembled using idba-ud. Assembled contigs were compared to the previous MAG obtained by MaxBin2 and if any improvement on the total assembly length, N50 and N90 statistics was observed, this new assembly was considered further, if no improvement was observed the original MaxBin2 Mollicute MAG was used instead. Final genome assemblies were then polished by SSPACE [80] with bwa as aligner and Ciclator 1.4.1 [81] was used to find possible circular scaffolds. After the ‘best’ assembly was obtained, manual curation of the contigs in each MAG was done by performing a BLASTn [82] search against the ‘nr’ database (downloaded on September 2016) and manually removing those contigs matching to insect or any other no Mollicutes bacteria (> 90% identity, <1e-5 e-value, > 80% coverage). Protein translated sequences of the three Mollicutes MAGs, one for each metagenome, were retrieved by Prodigal 2.6.3 [83]. The quality (completeness, contamination, and strain heterogeneity) of this manual curated Mollicutes MAG was then assessed by the checkM [79] pipeline using the protein predicted sequences from Prodigal and the following parameters: checkm lineage_wf --reduced_tree --genes -t 8 -x faa. Total genomic features prediction and open reading frame (ORFs) annotation on each MAGs were performed using PROKKA v1.11 [84] with the following parameters: --gcode 4 --metagenome --rfam. Metabolic pathways were predicted using the GhostKoala tool from KEGG [85] and manually curated using the Bioconductor R package KEGGREST 1.26.1 and the KEEG_parser tool developed for this work (https://github.com/avera1988/KEGG_parser). The dbCAN2 pipeline [86] was used to detect genes coding for putative carbohydrate-active enzymes (CAZymes) in the Mollicutes MAGs using the dbCAN-database 7 (downloaded in January 2019). Additionally, all Mollicutes MAGs were annotated using the NCBI Prokaryotic Genome Annotation Pipeline PGAP-4.6 [87] for public availability. Correlations between PROKKA, KEGG, CAZy and PGAP locus identifiers of each MAGs are shown in the Additional file 3 Data Set S2.

### Phylogenetic and comparative genomic analysis

Near-full-length (~ 1400-nucleotide [nt]) 16S rRNA gene sequences were retrieved from the annotations of the Mollicutes MAGs and compared to the NCBI “16S ribosomal RNA sequences” (accessed in February 2020) and Arb-SILVA (accessed in January 2020) databases using BLASTn to identify the nearest neighbors. MAFFT 7.3.10 [88] was used to align all sequences and a maximum-likelihood-based (ML) phylogenetic tree, based on the TVMe+R4 substitution model obtained by ModelFinder [89], was calculated by IQtree 1.6.12 [90] with 1000 Bootstrap replicates for internal branch support.

A total of 30 genomes (Additional file 2 Data set 1) across all the different *Spiroplasma* phylogenetic clades (i.e., Citri-Chrysopicola, Mirum, Apis, and Ixodetis) were downloaded from the NCBI “assembly” database (accessed in January 2020) and used for phylogenomic and comparative analyses. Pan and core genome analyses were conducted using GETHOMOLOGUES 2.0 [91], and OrthoMCL [92] was used for orthologue clustering (parameters: -A -c -t 0 -M -n 12). Single-copy genes from the core-genome of all *Spiroplasma* analyzed were parsed from the GETHOMOLOGUES pan-genome matrix results using custom Bash and Perl scripts (deposited in https://github.com/avera1988/Comparative_genomics).

For phylogenomics, a concatenated protein-based maximum-likelihood (ML) phylogenetic tree was constructed using the protein predicted sequences of 168 single-copy genes of *Spiroplasma* core genome. Protein sequences were concatenated and aligned using the multigenome2blocks pipeline [93]. ML phylogeny, based on the LG + F + R4 amino acid substitution model obtained by ModelFinder [89], was constructed using IQtree 1.6.12 [90] with 1000 Bootstrap replicates for internal branch support. Additionally, these 168 protein-coding genes were used to calculate the average amino acid identity (AAI) between *Spiroplasma* genomes using the AAI calculator from the “enveomics” collection tools [94]. AAI distance matrices were calculated and visualized by custom Perl and R scripts (deposited in https://github.com/avera1988/Comparative_genomics).

Finally, to compare functional profiles between *Dactylopius* associated and public *Spiroplasma*, all clusters of orthologous groups (COG) of proteins from each genome (Additional file 2 Data set 1) were annotated using the cdd2cog pipeline (https://github.com/aleimba/bac-genomics-scripts). Comparisons between proportions of COG genes from *Dactylopius* and non-*Dactylopius* associated *Spiroplasma* were performed using custom R scripts (deposited in https://github.com/avera1988/COG_differential_analysis). The number of genes on each COG category between *Dactylopius* and non-*Dactylopius*-associated *Spiroplasma* genomes were visualized by heatmaps in R 3.6.1.

### Presence of *S. ixodetis* in other *D. coccus* metagenomes from Mexico and Peru

Previously reported metagenomes of *D. coccus* collected from Mexico and Peru by Campana et al., [35] were used to detect the presence of *S. ixodetis* in different *Dactylopius* samples. Metagenomic reads were obtained by the fastq dump tool (https://github.com/ncbi/sra-tools) using the SRR1231828 and SRR1231831 accession numbers from the sequence-read archive (SRA) NCBI portal (https://www.ncbi.nlm.nih.gov/sra). Paired-reads of each metagenome (i.e., Mexico and Peru) were independently assembled using the idba-ud 11.1 [77] assembler with the default parameters. The assemblies resulted in 8085 (22,546,409 bp, N50 192,783 bp) and 10,617 (25,132,088 bp, N50 99,725 bp) contigs for the Mexican and Peruvian metagenomes, respectively. The resulting assembled metagenomic contigs were binned into MAGs by MaxBin 2.2.1 [78] with the following parameter -min_contig_length 900 for minimum contig length. The resulting MAGs were then classified by CheckM 1.1.2 pipeline [79] with the lineage_wf option for a broad taxonomical classification. In the case that no *Spiroplasma*-like MAGs were recovered, BLASTn searches were performed using as a database the index of *S. ixodetis* DCF genome and as query the total assembled contigs for each Mexican and Peruvian metagenomes with the following parameters: blastn -query contigs.fa -db DCF.fasta -max_target_seqs 1 -num_threads 8 -outfmt 6. Metagenomic contigs from both Mexican and Peruvian samples with an identity value > 90% and e-value < 0.0005 in the BLASTn searches were further classified as *S. ixodetis*-like contigs. To corroborate these contigs were taxonomically close to *Spiroplasma* spp. all *S. ixodetis*-like contigs recovered in each metagenome were annotated by BLASTx searches against the Uniref100 protein database (downloaded November 2020). Those contigs with a *Spiroplasma* match in the database were then scored as *S. ixodetis* contigs.

### Genes encoding components of the type IV secretion system (T4SS) detection

Protein sequence predictions from each *Spiroplasma* genome were compared to the type IV secretion system (T4SS) HMM profiles in the TXSSscan database [95] using the “hmmscan” (parameters: hmmscan –cpu 40 –domtblout) tool from HMMER 3.1b2 [96] to identify genes encoding domains that comprised potential components of T4SS. Putative T4SS component domains were manually parsed from hmmscan result tables and compared with previous PROKKA-PGAP annotations of each *Spiroplasma* genome.

### RNASeq and metatranscriptomic analysis

Metatranscriptomic data, previously obtained and reported by Bustamante-Brito et al., [31] from hemolymph, ovaries, and gut of female *D. coccus* cochineals, was used to analyze changes in the expression of *Spiroplasma* genes in different tissues of *Dactylopius*. Tissue dissection and RNA extraction procedures are fully described in [31]. Briefly, 30-s instar nymphs of *D. coccus* females were collected and superficially rinsed with ethanol 90% to remove the covering wax. Hemolymph was collected, pooled, and resuspended in 200 μl RNAlater (ThermoFisher) by doing a dorsal puncture in each insect with a 1 ml sterile syringe. After bleeding, all individuals were dissected under sterile conditions and the whole gut (including foregut, midgut, hindgut, and Malpighian tubules) and ovaries were independently collected, pooled, and resuspended in 200 μl of RNAlater (ThermoFisher). As for DNA extraction (see above), we tried to avoid tissue cross-contamination by rinsing the tissues several times with sterile phosphate-buffered saline. Total RNA was obtained from each pool of tissues (i.e., gut, hemolymph, and ovary) using the RNEasy kit (Qiagen) following the modification reported by Guerrero-Castro et al., [97]. High-quality RNA samples were used for cDNA strand-specific library preparation by the TruSeq Stranded Total RNA kit (Illumina), and ribosomal RNA (rRNA) present in the samples was removed with the RiboZero Removal kit for Bacteria (Illumina). Three biological replicates of each tissue were obtained, thus nine metatranscriptomic libraries were generated and sequenced using a single lane on an Illumina HiSeq4000 sequencer at Macrogen Korea, by a 100 bp read length pair-end protocol. Purity-filtered reads were adapter and quality (Phred score Q > 30) trimmed by TrimmGalore 0.4.4 pipeline with the --paired --q 30 options. Reads matching to rRNA sequences in each library were identified by Metaxa2 2.2–1 pipeline [98] using the -x T option and manually removed from the fastq files. To maintain read parity, all fastq files obtained after rRNA removal were subjected to a second round of filtering by TrimmGalore 0.4.4 with the --paired option and considered further.

### Differential gene expression analysis and quantification

To extract all the reads of *Spiroplasma* from the metatranscriptomes, high-quality filtered reads of each library (gut, hemolymph, and ovary) were independently aligned against the *Spiroplasma ixodetis* DCF genome using Hisat2 2.1.0 [99]. Properly mapped reads were obtained using samtools 1.7 with the following parameters: samtools fastq -@ 12 -c 6 -F 4 -N. The number of *Spiroplasma* read counts per gene locus in each library (tissue) was then obtained and summarized by the RSEM 1.3.1 pipeline [100] with the following parameters: rsem-calculate-expression -p 12 --paired-end --bowtie2 --estimate-rspd --append-names --output-genome-bam using the *S. ixodetis* DCF genomic annotation. Total matrix count with all abundances form each tissue replicate was obtained with the abundance_estimates_to_matrix.pl script from Trinity 2.4.0 [101] and parsed by the tximport Bioconductor package [102] in R. For differential expression analysis, treatments were classified depending on the RNAseq library as gut, hemolymph, and ovary. The software DESeq2 [103] was used to detect differentially expressed genes using as contrast each library/tissue comparison (i.e., gut vs hemolymph, gut vs ovary and hemolymph vs ovary). Genes were considered differentially expressed if the adjusted *p*-value (Benjamini-Hochber [BH] multiple test correction [104]) was less than or equal to 0.05 and an absolute fold-change was above 1.5 (absolute log_2_ fold-change ≥0.58) per each gene in any of the particular contrast comparison analyzed. Evaluation of main differences in expression per library (i.e., tissue) was assessed by a principal component analysis (PCA). PCA was performed and plotted by plotPCA function from DESeq2 with the variance stabilizing transformation (vst) method. Volcano plots and heatmaps were used to display and visualize all differential expressed genes of *S. ixodetis* DCF in the different libraries using the phamp and EnhancedVolcano (https://github.com/kevinblighe/EnhancedVolcano) packages in R. Fragments per kilobase of transcript per million mapped reads (FPKM) values of *S. ixodetis* DCF genes in each transcriptomic sample were obtained by the “fpkm” function of the DESeq2 package in R. All scripts to reproduce the gene expression analysis are deposited in Github (https://github.com/avera1988/Spiroplasma_ixodetis_Dactylopius_RNAseq).

## Supplementary Information

**Additional file 1: Figure S1.** Maximum-likelihood phylogenetic tree of the 16S rRNA from different Mollicutes. In red are the 16S rRNA sequences of *S. ixodetis* DO, DCM and DCF. Scale bar indicates 2% estimated sequence divergence. ModelFinder was used to calculate the TVMe+R4 nucleotide substitution model. Maximum-likelihood tree was constructed by IQTree with 1000 Bootstrap replicates for internal branch support. The 16S rRNA sequences of *Clostridioides difficile*, *Bacillus pumilus* and *Listeria innocua*, were used as outgroup. **Figure S2.** Maximum-likelihood phylogenetic tree of the virB4 ATPase coding gene from *S. ixodetis* DO, DCF and DCM (in red) and other organisms from the Genbank. Scale bar indicates 50% estimated sequence divergence. Accession numbers of all virB4 sequences are shown. MAFFT was used to align all sequences and a maximum-likelihood-based (ML) phylogenetic tree, based on the LG + I + G4 substitution model obtained by ModelFinder, was calculated by IQtree with 1000 Bootstrap replicates for internal branch support. **Figure S3.** Plasmid-like scaffolds encoding genes of the type IV secretion system (T4SS) in the *S. ixodetis* DO genome. Arrows represent the structure of the genes. The sequencing coverage per each scaffold is presented. **Figure S4.** Maximum-likelihood phylogenetic tree of plasmid (pink) and chromosomal (black) encoding virB4 ATPase of *S. ixodetisi* DO, DCM, DCF and plasmid encoding virB4 ATPase of *S. citri* and *S. kunkelii* (green). Scale bar indicates 50% estimated sequence divergence. Accession numbers of all *virB4* sequences are shown. MAFFT was used to align all sequences and a maximum-likelihood (ML) phylogenetic tree, based on the LG + F + G4 substitution model obtained by ModelFinder, was calculated by IQtree with 1000 Bootstrap replicates for internal branch support. **Figure S5.** General transcriptomic features of *S. ixodetis* DCF expressed genes in the gut ovary and hemolymph of *D. coccus.* (a) Number of RNAseq mapped reads to *S. ixodetis* DCF genome in different *Dactylopius* tissues. (b) Principal component analysis (PCA) after DESeq2 normalized transcripts. Colors correspond to different insect tissues: in green from gut (GUT), in orange from hemolymph (HM), and in purple from ovary (OV).

**Additional file 2: **. **Data set 1.** Genbank accession numbers of genomes and sequences used in this study and average amino acid identity (AAI) of *Spiroplasma* genomes.

**Additional file 3: **. **Data set 2.** General genome annotation and differential expression data of *S. ixodetis* from *Dactylopius*. DCF, DCM and DO sheets show all the annotations obtained from different programs and databases (i.e., Prokka, CAZyDB, COG, KEGG, NCBI-PGAP) of each genome. PlasmidLikeScaffolds sheet shows the gene features of putative plasmid-like sequences in DO, DCF and DCM genomes. COGCarbohydratemetabolism and COGAminoacidMetabolism sheets show the number of genes in each COG category annotated in DCF, DCM, DO genomes, and other 30 *Spiroplasma* spp. from the NCBI Genome database for the Carbohydrate transport and metabolism and Amino acid transport and metabolism categories, respectively. DifferentialExpressedGenes sheet show all the log2FoldChange, *p*-values, p-adjust values, and annotations of *S. ixodetis* DCF differential expressed genes in the gut, ovary and hemolymph tissues. Columns show the values of the different pair comparisons (i.e., gut vs hemolymph, gut vs ovary and hemolymph vs ovary). Expre.Values.w.AnnotationDCF shows all the DESeq2 results including the normalized expression as fragments per kilobase of transcript per million mapped reads (FPKM) values for each transcriptomic comparison and the annotation of all *S. ixodetis* DCF genes recovered.

## Data Availability

All metagenomic data from *D. coccus* females, *D. coccus* males and *D. opuntiae* was deposited with links to BioProject accession numbers PRJNA291435, PRJNA658779 and PRJNA658782 in the NCBI BioProject database (https://www.ncbi.nlm.nih.gov/bioproject/). Sequences of *S. ixodetis* DCF, DCM and DO genomes were deposited at DDBJ/ENA/GenBank under accession numbers JACSEQ000000000, JACSER000000000 and JACSES000000000, respectively. The genomic data has been deposited with links to BioProject accession number PRJNA655193 in the NCBI BioProject database (https://www.ncbi.nlm.nih.gov/bioproject/). *S. ixodetis* DCF raw metatranscriptomic reads from *D. coccus* gut, hemolymph and ovary were deposited in the NCBI Sequence Read Archive (SRA) with links to BioProject accession number PRJNA658344. A description of all software, including scripts and commands, used for the analyses in this paper can be found at https://github.com/avera1988.

## Abbreviations

T4SS: Type IV secretion system
ROS: Reactive oxygen species
RIP: Ribosome-inactivating proteins
Mbp: Mega base pairs
qRT-PCR: quantitative reverse transcriptase polymerase chain reaction
RNAseq: Ribonucleic acid sequencing
MAGs: Metagenome assembly genomes
CDS: Coding sequences
AAI: Average amino acid identity
COG: Clusters of orthologous groups of proteins
RNA: Ribonucleic acid
DNA: Deoxyribonucleic acid
KEGG: Kyoto encyclopedia of genes and genomes
PTS: Phosphotransferase system
PacBio: Pacific biosciences
BUSCO: Benchmarking Universal Single-Copy Orthologs
ML: Maximum-likelihood
FPKM: Fragments Per Kilobase of transcript per Million mapped reads
