# Supplementary material for: Functional genomics of a Spiroplasma associated with the carmine cochineals Dactylopius coccus and Dactylopius opuntiae
Source: BMC Genomics. 2021 Apr 6;22:240. doi: 10.1186/s12864-021-07540-2 (PMC8025503; doi:10.1186/s12864-021-07540-2)

Additional file 1. Figure S1. Maximum-likelihood phylogenetic tree of the 16S rRNA from different Mollicutes. In red are the 16S rRNA sequences of *S. ixodetis* DO, DCM and DCF. Scale bar indicates 2 % estimated sequence divergence. ModelFinder was used to calculate the TVMe+R4 nucleotide substitution model. Maximum-likelihood tree was constructed by IQTree with 1000 Bootstrap replicates for internal branch support. The 16S rRNA sequences of *Clostridioides difficile*, *Bacillus pumilus* and *Listeria innocua*, were used as outgroup.

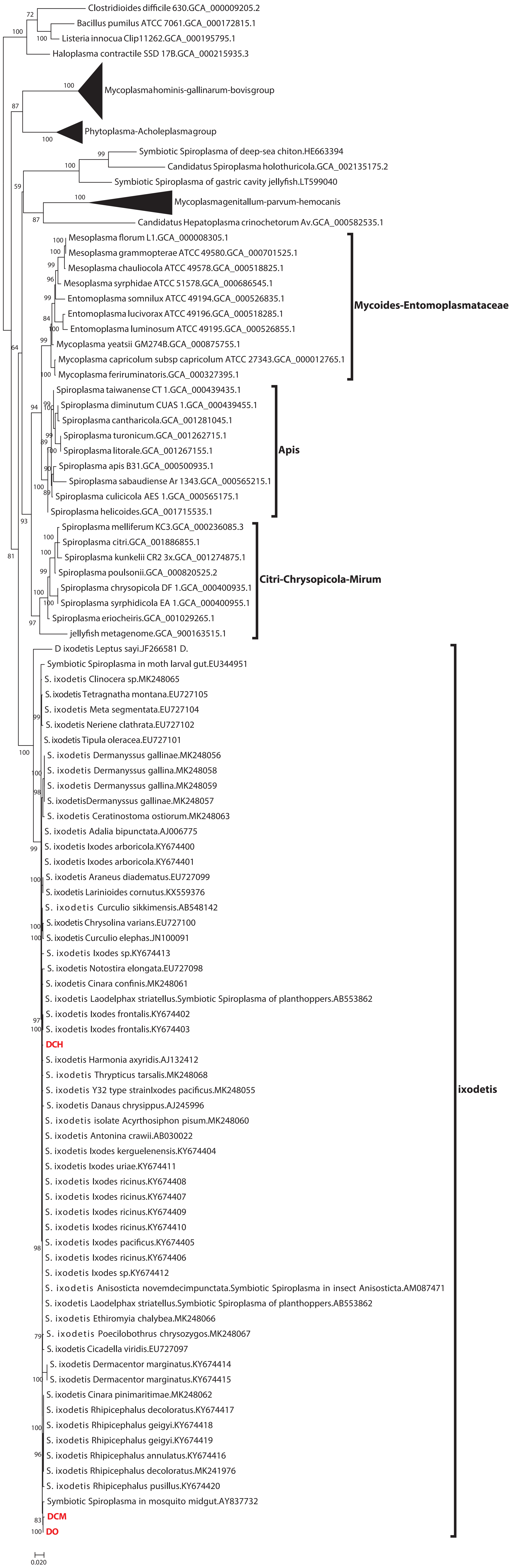

Additional file 1. Figure S2. Maximum-likelihood phylogenetic tree of the virB4 ATPase coding gene from *S. ixodetis* DO, DCF and DCM (in red) and other organisms from the Genbank. Scale bar indicates 50 % estimated sequence divergence. Accession numbers of all virB4 sequences are shown. MAFFT was used to align all sequences and a maximum-likelihood-based (ML) phylogenetic tree, based on the LG+I+G4 substitution model obtained by ModelFinder, was calculated by IQtree with 1000 Bootstrap replicates for internal branch support.

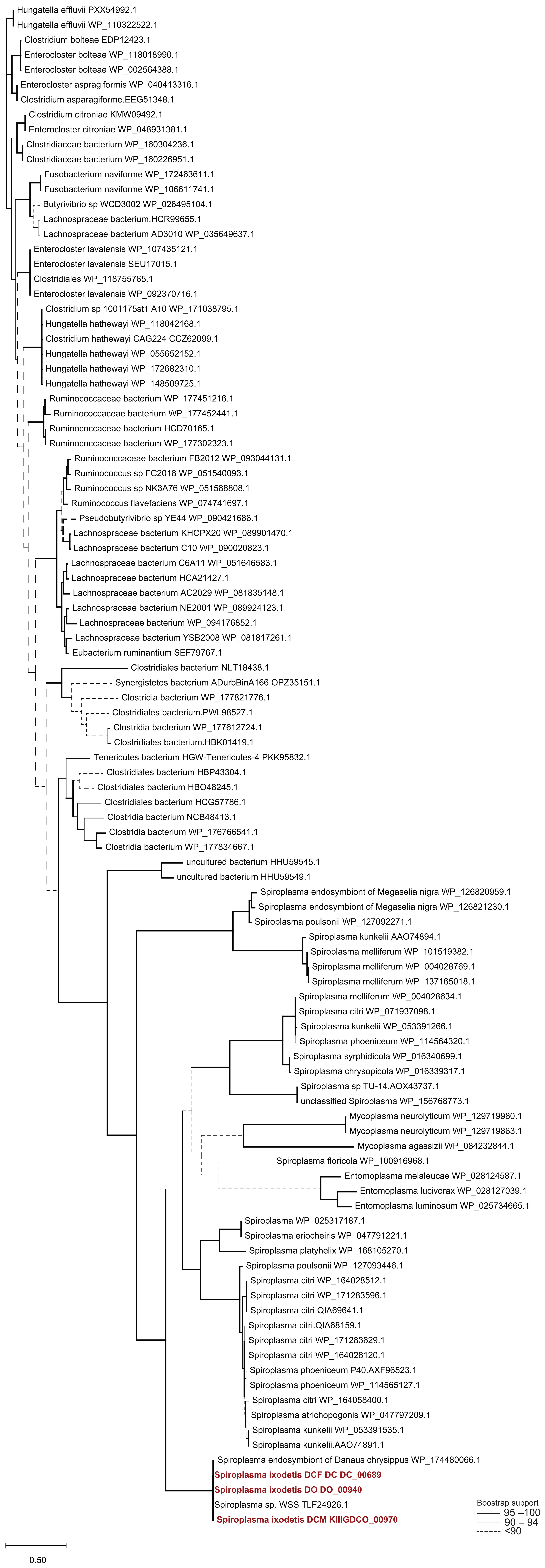

Additional file 1. Figure S3. Plasmid-like scaffolds encoding genes of the type IV secretion system (T4SS) in the *S. ixodetis* DO genome. Arrows represent the structure of the genes. The sequencing coverage per each scaffold is presented.

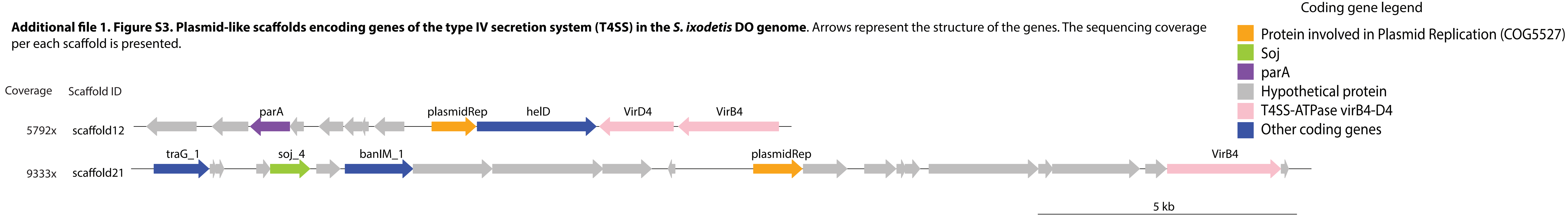

Additional file 1. Figure S4. Maximum-likelihood phylogenetic tree of plasmid (pink) and chromosomal (black) encoding virB4 ATPase of *S. ixodetis* DO, DCM, DCF and plasmid encoding virB4 ATPase of *S. citri* and *S. kunkelii* (green). Scale bar indicates 50 % estimated sequence divergence. Accession numbers of all *virB4* sequences are shown. MAFFT was used to align all sequences and a maximum-likelihood (ML) phylogenetic tree, based on the LG+F+G4 substitution model obtained by ModelFinder, was calculated by IQtree with 1000 Bootstrap replicates for internal branch support.

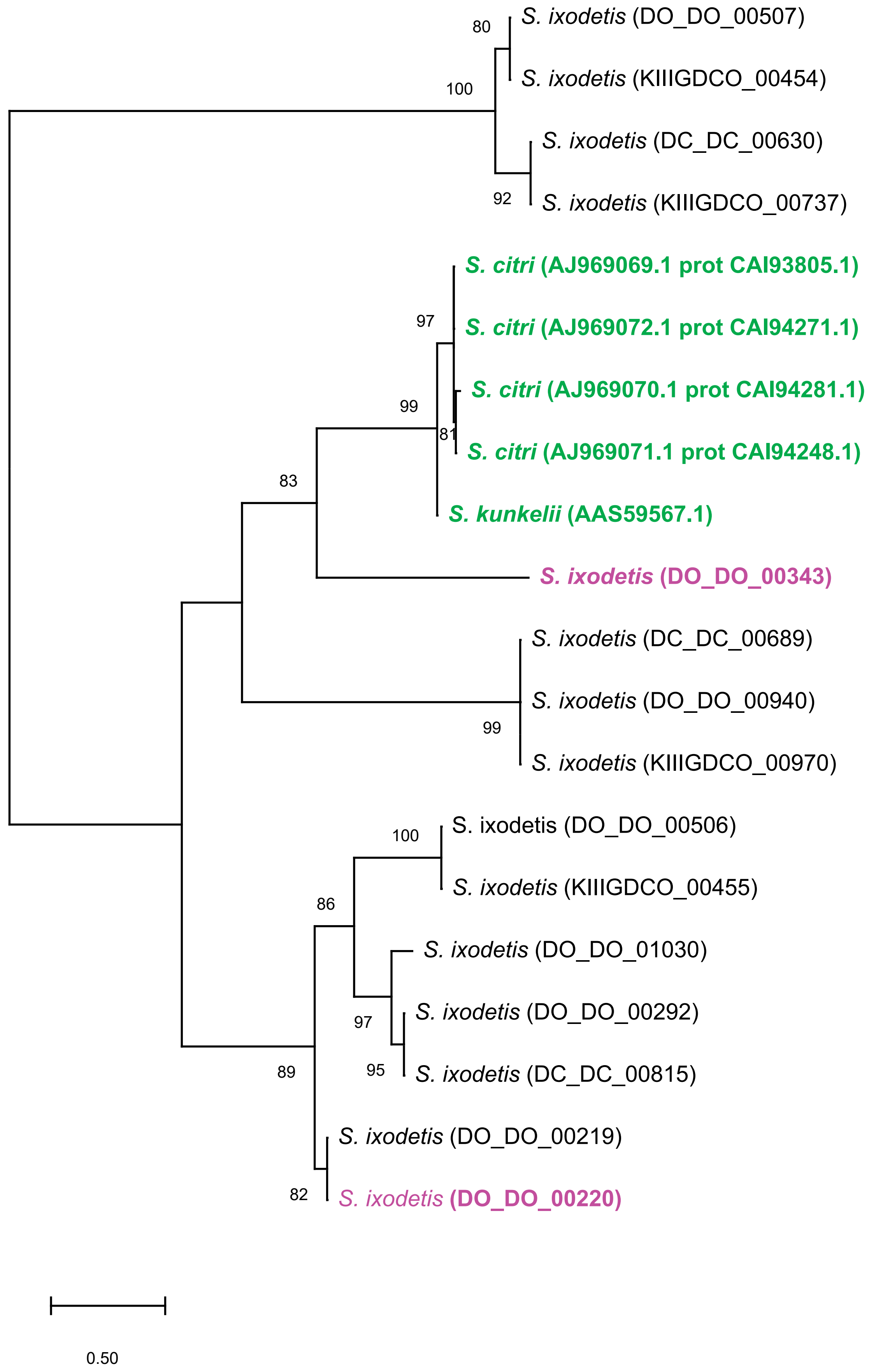

Additional file 1. Figure S5. General transcriptomic features of *S. ixodetis* DCF expressed genes in the gut ovary and hemolymph of *D. coccus*. (a) Number of RNAseq mapped reads to *S. ixodetis* DCF genome in different *Dactylopius* tissues. (b) Principal component analysis (PCA) after DESeq2 normalized transcripts. Colors correspond to different insect tissues: in green from gut (GUT), in orange from hemolymph (HM), and in purple from ovary (OV).

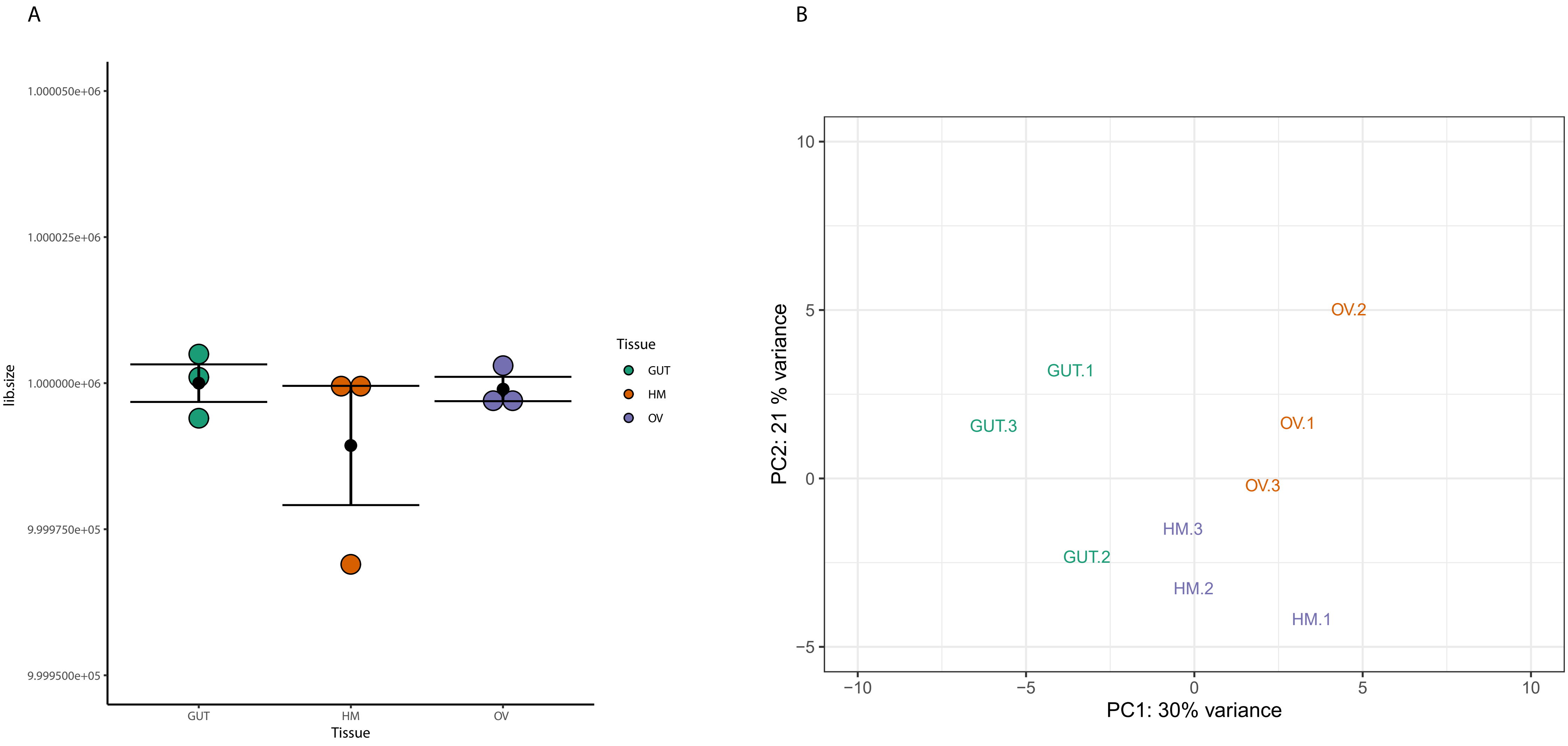

Supplement: Supplementary file 1 — Additional file 1: Figure S1. Maximum-likelihood phylogenetic tree of the 16S rRNA from different Mollicutes. In red are the 16S rRNA sequences of S. ixodetis DO, DCM and DCF. Scale bar indicates 2% estimated sequence divergence. ModelFinder was used to calculate the TVMe+R4 nucleotide substitution model. Maximum-likelihood tree was constructed by IQTree with 1000 Bootstrap replicates for internal branch support. The 16S rRNA sequences of Clostridioides difficile, Bacillus pumilus and Listeria innocua, were used as outgroup. Figure S2. Maximum-likelihood phylogenetic tree of the virB4 ATPase coding gene from S. ixodetis DO, DCF and DCM (in red) and other organisms from the Genbank. Scale bar indicates 50% estimated sequence divergence. Accession numbers of all virB4 sequences are shown. MAFFT was used to align all sequences and a maximum-likelihood-based (ML) phylogenetic tree, based on the LG + I + G4 substitution model obtained by ModelFinder, was calculated by IQtree with 1000 Bootstrap replicates for internal branch support. Figure S3. Plasmid-like scaffolds encoding genes of the type IV secretion system (T4SS) in the S. ixodetis DO genome. Arrows represent the structure of the genes. The sequencing coverage per each scaffold is presented. Figure S4. Maximum-likelihood phylogenetic tree of plasmid (pink) and chromosomal (black) encoding virB4 ATPase of S. ixodetisi DO, DCM, DCF and plasmid encoding virB4 ATPase of S. citri and S. kunkelii (green). Scale bar indicates 50% estimated sequence divergence. Accession numbers of all virB4 sequences are shown. MAFFT was used to align all sequences and a maximum-likelihood (ML) phylogenetic tree, based on the LG + F + G4 substitution model obtained by ModelFinder, was calculated by IQtree with 1000 Bootstrap replicates for internal branch support. Figure S5. General transcriptomic features of S. ixodetis DCF expressed genes in the gut ovary and hemolymph of D. coccus. (a) Number of RNAseq mapped reads to S. ixodetis DCF [file 12864_2021_7540_MOESM1_ESM.pdf]
